# Phasic Contractions of the Mouse Vagina and Cervix at Different Phases of the Estrus Cycle and during Late Pregnancy

**DOI:** 10.1371/journal.pone.0111307

**Published:** 2014-10-22

**Authors:** Fernanda S. Gravina, Dirk F. van Helden, Karen P. Kerr, Ramatis B. de Oliveira, Phillip Jobling

**Affiliations:** School of Biomedical Sciences & Pharmacy, Faculty of Health & Medicine, The University of Newcastle, Callaghan, NSW, Australia; Cinvestav-IPN, Mexico

## Abstract

**Background/Aims:**

The pacemaker mechanisms activating phasic contractions of vaginal and cervical smooth muscle remain poorly understood. Here, we investigate properties of pacemaking in vaginal and cervical tissues by determining whether: 1) functional pacemaking is dependent on the phase of the estrus cycle or pregnancy; 2) pacemaking involves Ca^2+^ release from sarcoplasmic/endoplasmic reticulum Ca^2+^-ATPase (SERCA) -dependent intracellular Ca^2+^ stores; and 3) c-Kit and/or vimentin immunoreactive ICs have a role in pacemaking.

**Methodology/Principal Findings:**

Vaginal and cervical contractions were measured *in vitro*, as was the distribution of c-Kit and vimentin positive interstitial cells (ICs). Cervical smooth muscle was spontaneously active in estrus and metestrus but quiescent during proestrus and diestrus. Vaginal smooth muscle was normally quiescent but exhibited phasic contractions in the presence of oxytocin or the K^+^ channel blocker tetraethylammonium (TEA) chloride. Spontaneous contractions in the cervix and TEA-induced phasic contractions in the vagina persisted in the presence of cyclopiazonic acid (CPA), a blocker of the SERCA that refills intracellular SR Ca^2+^ stores, but were inhibited in low Ca^2+^ solution or in the presence of nifedipine, an inhibitor of L-type Ca^2+^channels. ICs were found in small numbers in the mouse cervix but not in the vagina.

**Conclusions/Significance:**

Cervical smooth muscle strips taken from mice in estrus, metestrus or late pregnancy were generally spontaneously active. Vaginal smooth muscle strips were normally quiescent but could be induced to exhibit phasic contractions independent on phase of the estrus cycle or late pregnancy. Spontaneous cervical or TEA-induced vaginal phasic contractions were not mediated by ICs or intracellular Ca^2+^ stores. Given that vaginal smooth muscle is normally quiescent then it is likely that increases in hormones such as oxytocin, as might occur through sexual stimulation, enhance the effectiveness of such pacemaking until phasic contractile activity emerges.

## Introduction

The female reproductive tract (FRT) is comprised of a series of embryologically related, yet specialized organs that coordinate sexual activity, conception, fetal growth and parturition. Many of these functions depend on the coordinated contraction of smooth muscle. Motility along the length of the FRT is coordinated by a combination of phasic contractile activity and nervous control, all strongly influenced by hormones. Most research to date has concentrated on uterine motility especially with a view to understanding pregnancy and development of strategies for the prevention of pre-term birth. In contrast, the role of vaginal or cervical motility has received less attention [1], particularly in terms of the drivers of phasic contractile activity.

It is widely established that pacemaking in the gastrointestinal tract is driven by interstitial cells of Cajal (ICCs). These form extensive networks within the gut wall where they are strongly electrically coupled to each other and to smooth muscle. ICCs generate oscillations of membrane potential in smooth muscle known as “slow waves” that subserve a key role in gastrointestinal peristalsis [2], [3], [4], [5]. Interstitial cells (ICs) within other tissues are generally identified in the same way as ICCs by the expression of CD117/c-Kit and/or vimentin [6]. Cells immunoreactive for c-Kit and vimentin have been found in the uterus and human vagina [7], [8] leading to speculation that pacemaking in the FRT is driven by similar mechanisms to that in the gut [9].

It remains unknown, however, whether ICs are pacemaker cells in the FRT. An electrophysiological study of isolated uterine ICs failed to find the inward ionic currents, which are critical for pacemaking [8]. Furthermore, prolonged inhibition of the c-Kit receptor has variable effects on uterine pacemaking yet consistently disrupts gastrointestinal pacemaking [9], [10], [11], [12]. Various studies indicate Ca^2+^ stores do not have a role in uterine pacemaking [13], [14], [15], [16] but there have been contrary views [17] with c-Kit positive cells suggested as the protagonists [9], [12], [18], though a more recent study in human myometrium by the Popescu group indicates that ICs which they term telocytes were found to modulate rather than generate contractions [19].

The pacemaker mechanism in classical ICCs in the GIT is inhibited by blockers of the sarcoplasmic/endoplasmic reticulum Ca^2+^-ATPase (SERCA) and hence of intracellular ER/SR Ca^2+^ stores, but not affected by blockade of extracellular Ca^2+^ through L-Type Ca^2+^ channels [20], [21], [22]. While knowledge on uterine pacemaking is accumulating, less is known about smooth muscle pacemaker activity in the cervix and vagina. The cervix is often considered to be part of the uterus, however it is histologically and functionally distinct. It undergoes biochemical changes during pregnancy, when it softens and dilates at term [23]. The cervix while having some smooth muscle is primarily composed of collagen [24] and for this reason few studies have evaluated contractility. This tissue, however, is able to contract spontaneously in sheep [25] and cow [26]. Studies in the mouse have generally focused on investigating cervical ripening [27] but not cervical contraction, as studied here.

The mechanisms underlying motility in the vagina are poorly understood. In women, intravaginal pressure rises during sexual activity and rhythmic pressure waves accompany orgasm [28]. Much of this rhythmic activity, however, seems to be driven by circumvaginal striated muscle and not vaginal smooth muscle [28], [29]. Vaginal smooth muscle appears to undergo sustained contraction seen as a rise in baseline pressure [29]. Subsequent work has demonstrated electrical waves and concomitant pressure changes in human vagina [30]. Importantly these were abolished by intravaginal anaesthesia suggesting a nervous component.

Animal models have also been used for the study of vaginal function during sexual behaviours [31], [32]. Initiation of the urethrogenital reflex leads to phasic vaginal contractions [1]. As with humans the mechanisms driving these phasic vaginal contractions are unclear and it is not known if they are due to pacemaking within the smooth muscle layer or are driven by autonomic nervous activity.

The vagina is densely innervated [33], [34] and the smooth muscle in its wall contracts under electrical field stimulation *in vitro* [35]. The contribution of the smooth muscle to fertility and parturition is yet to be fully clarified though it has been speculated that during the female orgasm that vaginal pressure may play a role in transporting semen into the cervix [36].

In this study we investigated spontaneous or induced phasic contractions in the cervix and vagina during the different phases of the estrus cycle and in late pregnancy comparing our findings to corresponding uterine activity.

## Materials and Methods

### Ethics Statement

All experiments were approved by the University of Newcastle Animal Care and Ethics Committee for ethics approval A-2009-153 according to the Australian Code of Practice for the care and use of animals for scientific purposes as released by the National Health and Medical Research Council of Australia in 2004.

### Animals and tissue collection

Late pregnant (17–19 days post fertilization) and young (2 months) adult non-pregnant female Swiss mice were maintained in a controlled environment in a 12/12 h light/dark cycle, at a temperature of approximately 20°C, with free access to food and water. Animals were euthanized by inducing deep anaesthesia with isoflurane inhalation (5–10% in the air) followed by exsanguination. Vaginal smears using 0.9% sodium chloride and methylene blue were taken in order to identify the stage of the estrous cycle in non-pregnant mice. The reproductive tract was then dissected out and placed in physiological saline solution (PSS) of composition (mM): NaCl 120, KCl 5, CaCl_2_ 2.5, MgCl_2_ 2, NaHCO_3_ 25, NaH_2_PO_4_ 1, glucose 10 and bubbled with carbogen (95% O_2_, 5% CO_2_).

### Immunohistochemistry

For determination of smooth muscle actin, samples of uterus, cervix and vagina were prepared and fixed in 4% paraformaldehyde overnight. After fixation, tissues were washed with 80% ethanol, dimethylsulfoxide and dehydrated in 100% ethanol. They were placed in melted polyethylene glycol (PEG, MW 1000) for 2 h at 46°C and then embedded in melted PEG (MW 1450) in a cryostat mould and frozen. Tissues were sectioned at 15 μm using a standard bench microtome (American Optical). Sections were incubated in monoclonal anti-α-smooth muscle actin Cy3 conjugated (1∶500 clone 1A4, Sigma, St Louis). Following this, sections were washed in PBS, mounted on slides in carbonate-buffered glycerol (pH 8.6) and examined under an Olympus BX51 microscope with appropriate filters.

Labelling for ICs was performed using whole-mount preparations of reproductive tract wall. Samples of approximately 5×5 mm were cut, fixed in either acetone (for subsequent c-Kit labelling) or 4% paraformaldehyde for visualization of vimentin. Rat anti-c-Kit CD117 (Chemicon/Millipore, MA) antiserum was used at a dilution of 1∶500. Goat anti-vimentin (Sigma, St Louis) was used at a concentration of 1∶50. Anti-c-Kit CD117 was visualized with donkey anti-rat fluorescein isothiocyanate (FITC) and vimentin was visualized with donkey anti-goat FITC (Jackson ImmunoResearch, West Grove, PA). Labelled cell densities were quantified from confocal sections of wholemounts using a 60x objective and 212×212 μm fields of view.

### Contractility

Strips of cervix and vagina prepared from circumferentially oriented smooth muscle with mucosa left attached were trimmed of connective tissue and mounted in 3 ml organ baths. These, together with longitudinal uterine strips with endometrium left attached, were equilibrated under 5 mN for 10 min in physiological saline solution (PSS) at 37°C containing (mM): NaCl 120, KCl 5, CaCl_2_ 2.5, MgCl_2_ 2, NaHCO_3_ 25, NaH_2_PO_4_ 1, glucose 10 and bubbled with carbogen (95% O_2_, 5% CO_2_). For Ca^2+^ free PSS, CaCl_2_ was substituted by equimolar MgCl_2_ with 0.5 mM EGTA added. KCl (40 mM) added to PSS was applied for a short time (∼2 min) to confirm tissue viability. Spontaneous or agonist-induced contractions were recorded isometrically via a Grass FT03C tension transducer connected to a MacLab 4e recording system. Tissues that exhibited spontaneous contractions tended to become active within 30 min and then maintain contractions for the next 1.5 hours although there was some variability in this activity. Experiments generally involved equilibration for 30 minutes followed by 30 min monitoring of control contractions and then 30 min application of test solution. Measurements were generally made using the last 5 minutes of activity in control and test solutions.

### Chemicals

Oxytocin, tetraethylammonium chloride (TEA), cyclopiazonic acid (CPA) and nifedipine were obtained from Sigma (St Louis, MO). Oxytocin and TEA were dissolved in water. CPA and nifedipine were dissolved in dimethyl sulphoxide (DMSO). The final concentration of DMSO in the PSS did not exceed 1∶1000.

### Statistics

Statistical analysis involved using one-way ANOVA with Tukey post hoc test, a paired *t-*test or Wilcoxon matched-pairs signed rank test for paired data (depending on the normality of the data as determined using the Kolmogorov–Smirnov test). Data is presented as the mean ± SE (standard error of the mean) with * indicating significance at *P*<0.05.

### Main Outcome Measures

The strength and frequency of spontaneous and induced phasic contractions, as studied *in vitro* was compared in the uterus, cervix and vagina. Immunofluorescence was used to test for the presence of c-Kit or vimentin positive ICs. Repeat experiments were made on tissues from different animals, so “n” refers to both the number of tissues and animals.

## Results

### Distribution of smooth muscle

**Figure 1:**
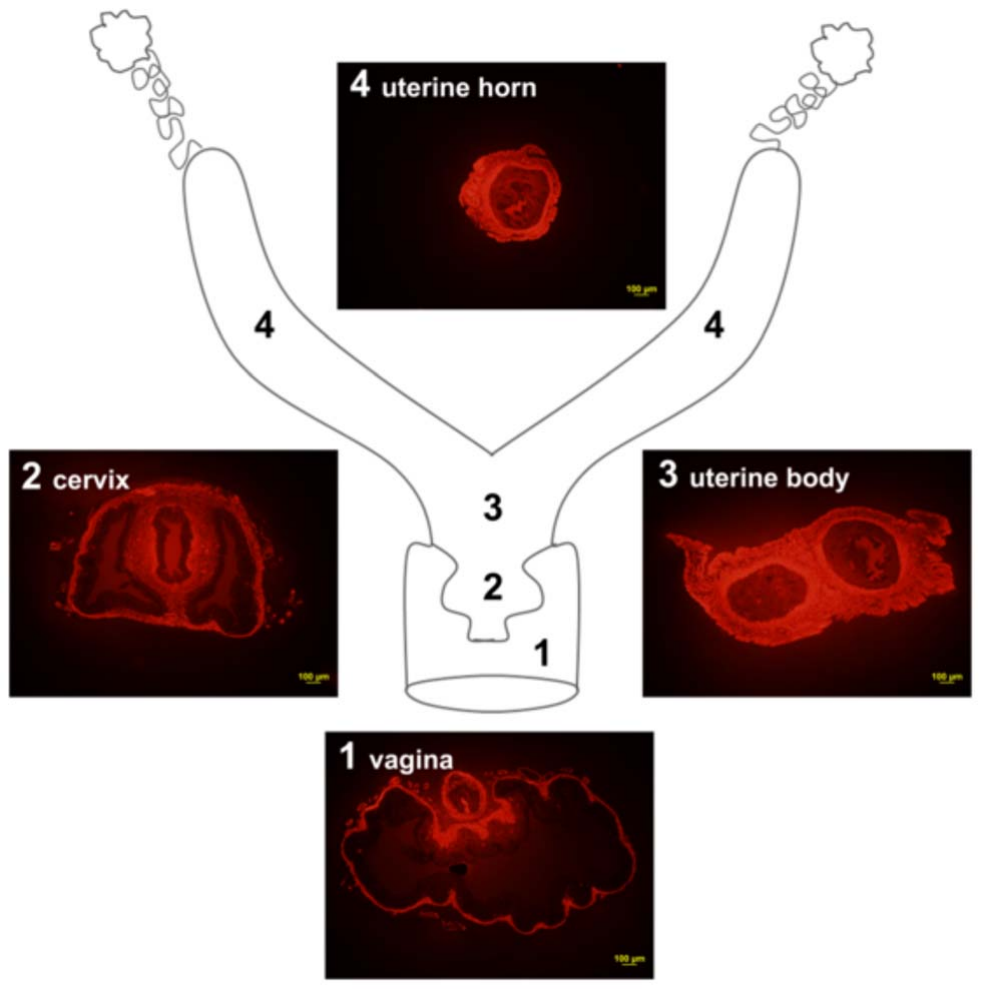
Anatomy of the mouse female reproductive tract. The diagram shows the regions at which the corresponding sections, labeled with smooth muscle α-actin conjugated to Cy3, were obtained (1 – vagina, 2 – cervix with surrounding vaginal tissue, **3** – uterine body, **4** – uterine horn). The uterine body and horn present a denser and wider muscle layer than the cervix and vagina.

Transverse histological sections of the female mouse reproductive tract in the Swiss mice of our studies (Figure 1) indicate that 72±13%, 45±9% and 29±10% (n = 3 for each) of the wall thickness in uterus, vagina & cervix is made up of smooth muscle, respectively.

### Spontaneous and induced contractions

**Figure 2:**
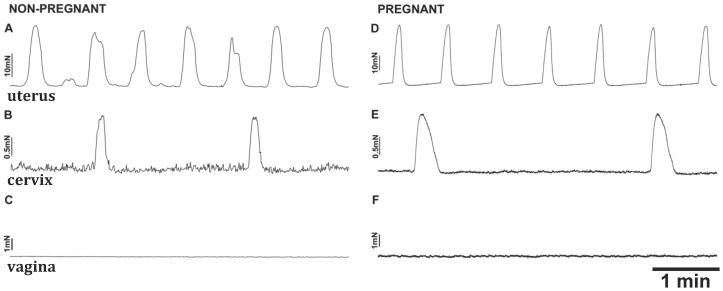
Tension recordings from cervical and vaginal strips of smooth muscle held at 5 mN resting tension in PSS with uterine data obtained from longitudinal muscle strips presented for comparative purposes. Non-pregnant (**A**) and late pregnant (**D**) mice uteri exhibited spontaneous contractions. The cervices from non-pregnant mice (**B**) exhibited spontaneous contractions in estrus and metestrus, but not diestrus and proestrus (see figure 3 for more details); while cervices from pregnant mice (**E**) always exhibited spontaneous contractions. Vaginal tissues from non-pregnant (**C**) and pregnant (**F**) mice were generally not spontaneously active.

Cervical tissues from nearly half the non-pregnant mice were spontaneously active (47% active, n = 17, Figure 2B), while cervical tissues from all late pregnant mice showed spontaneous contractions (n = 48, Figure 2E). By comparison, uterine tissues always exhibited spontaneous contractions, when obtained from either non-pregnant (n = 17, Figure 2A) or late pregnant mice (19 days gestation, n = 9, Figure 2D). Contractions in both the active cervix and uterus were reasonably stable, the 60/30 min frequency and amplitude ratios (5 min sampling) being 0.76±0.12 and 0.78±0.24 (n = 3) respectively for the cervix and 0.82±0.15 and 0.93±0.17 (n = 7) respectively for the uterus. Uterine tissues obtained from mice in middle pregnancy (14 days) were quiescent (n = 2, data not shown). Vaginal tissues were almost never spontaneously active (1 of 22 vaginal strips, Figure 2C, F).

**Figure 3:**
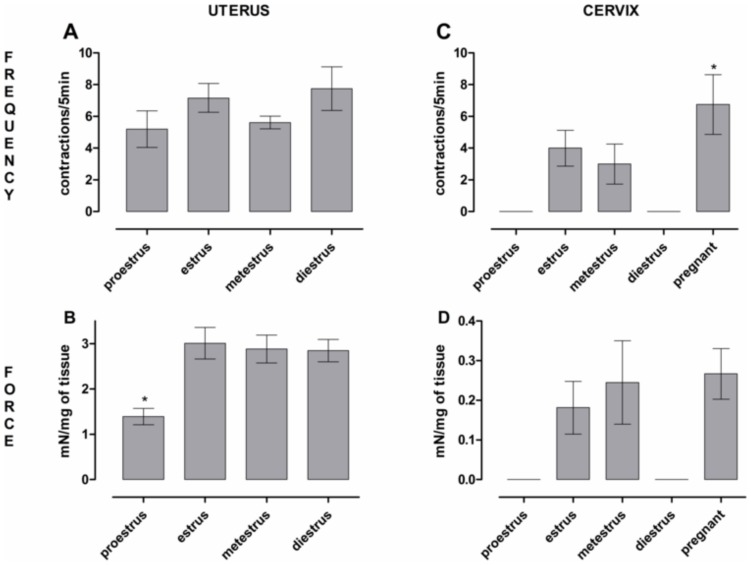
The effect of the oestrus cycle and late pregnancy on uterine and cervical spontaneous activity. **A**& **C**, Frequency of uterine and cervical contractions (per 5 min), indicating that the uterus is spontaneously active throughout the oestrus cycle and the cervix is spontaneously active in estrus, metestrus and in pregnant mice; * mean frequency of cervices from pregnant mice is slightly higher than estrus and metestrus (* *P*<0.05). This difference is small and has not been further investigated. **B** & **D**, the force of uterine and cervical contractions in the different stages of the oestrus cycle and in pregnancy. While significant (* *P*<0.05), the difference in contraction strength for uterine tisues at proestrus has not been further investigated. Data is presented as the mean ± SE with n = 4–9.

The finding that only about half of cervices from non-pregnant mice were active, led us to investigate the relation between activity and the estrous cycle. Cervical tissues were quiescent during proestrus (n = 3) and in 3 of 4 cases during diestrus, but spontaneously active in 5 of 6 cases during estrus, in 6 of 8 cases during metestrus and in all 9 cases in tissues from late pregnant mice (Figure 3C). The small but significant difference in frequency of cervical tissues from pregnant mice has not been further investigated. Cervical contractions during the most active phases (estrus and metestrus) were of similar force (when corrected by tissue weight) to that measured in cervical tissues from late pregnant mice (Figure 3D). The frequency of uterine contractions was not significantly different through the phases of the cycle and in late pregnancy (Figure 3A). However, the force of uterine contractions was weaker in proestrus when compared to the other phases of the estrous cycle and to late pregnancy (Figure 3B). This difference in contraction strength for uterine tissues has not been further investigated.

### Agonist-induced contractions

**Figure 4:**
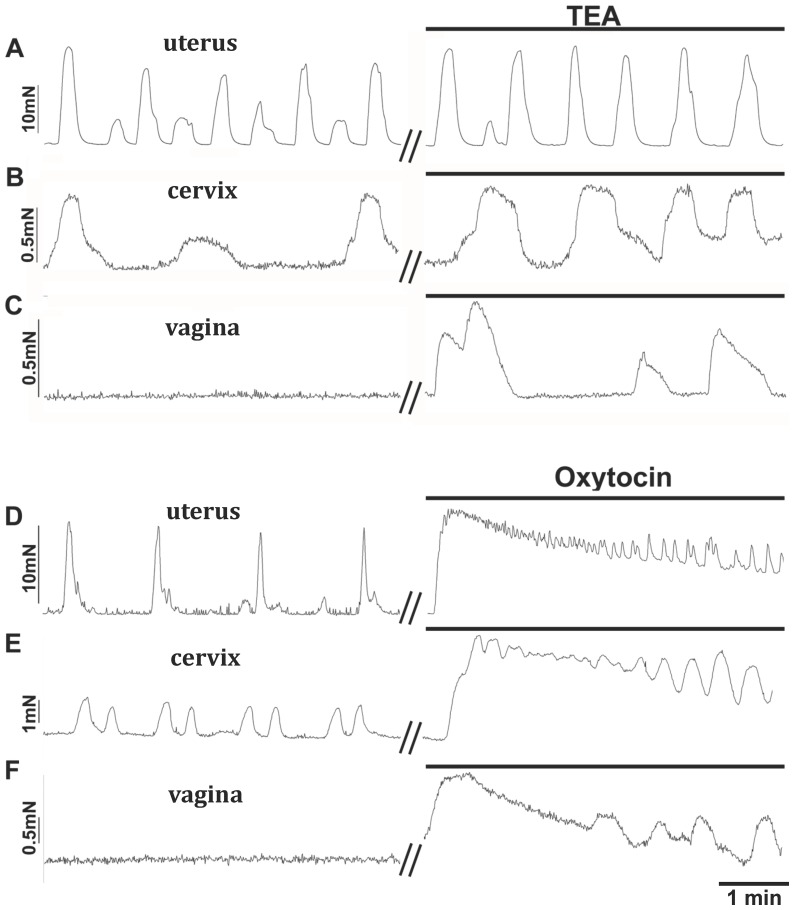
Contractile responses recorded in response to the potassium channel blocker tetraethylammonium chloride (TEA) or an agonist (oxytocin) on cervical and vaginal smooth muscle with uterine data presented for comparative purposes. Application of TEA (10 mM) enhanced spontaneous contractions in uterine (**A**) and cervical (**B**) tissues and caused vaginal (**C**) and cervical tissues in diestrus and proestrus (data not shown) to become spontaneously active. Application of oxytocin (1 nM) to uterine (**D**), cervical (**E**) and vaginal (**F**) tissues caused a large contraction that gradually decreased revealing spontaneous contractions at high frequency for all three tissues.

In order to see if vaginal muscle could be induced to exhibit phasic contractions we tested the K^+^ channel blocker TEA and oxytocin. TEA is known to increase contractility in rat myometrial strips through increasing the excitability of smooth muscle [37] most likely by blocking voltage dependent K^+^ channels such as Kv7, inhibition of which has been shown to increase myometrial contractions [38]. Oxytocin, which is known to enhance spontaneous uterine contractions, induced phasic vaginal contractions. For comparison, we also tested TEA and oxytocin on the cervix and uterus. TEA increased the strength of uterine and cervical contractions (Figure 4A, B, n = 4 for both). Notably, it induced 19/22 normally quiescent vaginal tissues to exhibit strong phasic contractions of amplitude near 0.2 mN/mg tissue weight at a frequency of 7.1±1.7 per 5 min (Figure 4C; see also [39]). TEA-induced contractions were reasonably stable over the time period of our experiments, the 60/30 min frequency and amplitude ratios being 0.82±0.19 and 0.84±0.11 (n = 5) respectively. Oxytocin (1 nM) caused tonic contractions of the uterine, cervical and vaginal smooth muscles that gradually declined in amplitude with the onset of phasic contractions (Figure 4D–F; n = 5, 5 & 4 respectively).

### C-Kit and vimentin immunoreactive cells

**Figure 5:**
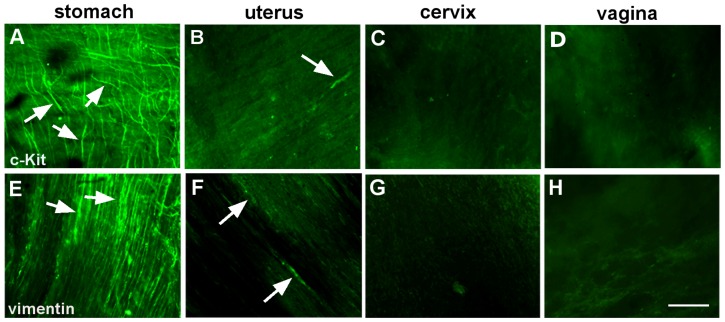
Expression of c-Kit or vimentin immunoreactivity in the reproductive tract. Single confocal optical sections taken from wholemount preparations. As previously reported (Duquette *et al*., 2005) c-Kit or vimentin immunoreactive cells are present in the myometrium (white arrows) (**B** & **F**). However these were rarely observed in the cervix (**C** & **G**) and not observed in the vagina (**D** & **H**). Studies made for comparative purposes under the same conditions show the well reported extensive network of ICCs (white arrows) present in the stomach (**A** & **E**). Scale bar  = 100µm.

We next investigated the presence of c-Kit or vimentin immunoreactive ICs in cervical and vaginal wholemounts. C-Kit-IR cells and vimentin-IR cells were very sparse in the cervix (0.5±0.3, n = 5 and 2.7±0.9, n = 3 cells per field of view; Figure 5C, G). We found no c-Kit-IR (n = 4) or vimentin-IR cells (n = 3) in the vagina (Figure 5D, H). As a positive control, and as has been previously demonstrated [7] (Duquette, Shmygol et al. 2005) c-Kit and vimentin-IR cells were present in the uterus. In our mice, we found 1.2±0.2 (n = 5) c-Kit-IR and 5.7±1.8 (n = 3) vimentin-IR cells labelled per field of view in uterine wholemounts. Importantly these cells did not obviously form a network (Figure 5B, F), unlike the dense networks formed by gastric ICCs from the proximal gastric antrum (Figure 5A, E; also see [40]).

### SERCA-dependent Ca^2+^ stores

**Figure 6:**
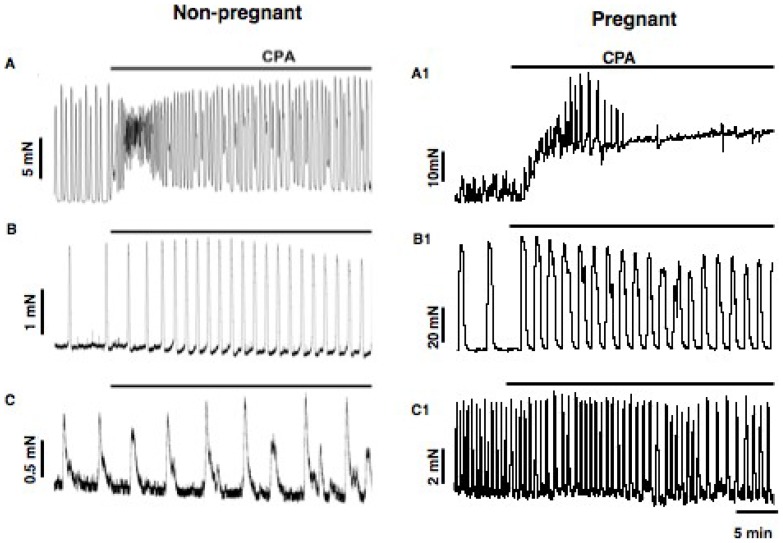
Spontaneous contractions occur in the presence of CPA, an inhibitor of SR Ca^2+^ stores. Shown are the effects of inhibiting the SERCA using 10 µM CPA on contractions in uterine (**A, A1**), cervical (**B, B1**) and vaginal (**C, C1**) smooth muscle strips from non-prenant and pregnant mice. Recordings from vaginal tissues were made in the presence of TEA (10 mM) to reveal contractions. CPA (10 µM) applied for at least 30 minutes modulated but did not abolish the contractions in any of the three tissue types.

The effect of the SERCA inhibitor CPA (10 µM) on spontaneous cervical and 10 mM TEA-induced phasic vaginal contractions was investigated. CPA caused a 2-fold increase in cervical contraction frequency in tissues from non-pregnant mice (4.5±0.9 vs. 2.1±1.0 contractions/5 min, n = 4, *P*<0.05) but did not significantly alter cervical contraction frequency in tissues from pregnant mice (n = 5). CPA did not significantly alter the TEA-induced vaginal contraction frequency in tissues from non-pregnant and pregnant mice (n = 4 & 5 respectively). CPA did not cause a significant increase in tone in these tissues. By comparison and as is known [13], [14], [15], [16], [39], application of CPA to uterine tissues from non-pregnant and pregnant mice caused an increase in resting tone and contraction frequency especially during the period shortly after application (Fig. 6A; n = 4). Notably, uterine contractions persisted during CPA application in tissues from non-pregnant mice but were abolished in uteri from pregnant mice (Fig. 6A1; n = 5).

The role of Ca^2+^ on spontaneous contractions was examined by reducing extracellular Ca^2+^ entry by bathing the tissues in nominally Ca^2+^ free solution or blocking L-type Ca^2+^ channels with nifedipine. Cervical and 10 mM TEA-induced vaginal contractions, as studied in non-pregnant mice, were abolished in the presence of nominally Ca^2+^-free solution (n = 4 for each tissue type) or in the presence of 2 µM nifedipine (n = 4 for each tissue type). As previously reported [39], [41], low Ca^2+^ solution and nifedipine also abolished uterine contractions (n = 4).

## Discussion

### Spontaneous activity along the FRT

Cervices were spontaneously contractile when taken from mice in late pregnancy or in most cases from non-pregnant mice during, estrus and metestrus but not during proestrus and diestrus. Progesterone, which is known to inhibit uterine contractions [42], is high in proestrus with levels drastically dropping in estrus. In metestrus, levels of progesterone start to rise slowly, reaching high concentration during diestrus [43]. Pregnancy quiescence is primarily maintained by high levels of progesterone, with levels decreasing towards birth [44], including near-term pregnant mice, as used in this study. Therefore, it is believed that high levels of progesterone within the tissue would inhibit cervical contractions, while low levels (estrus, metestrus and late pregnancy) allow spontaneous contractions. In contrast, uterine tissues were always spontaneously active when taken from mice in late pregnancy or from non-pregnant mice during all stages of the estrus cycle (see also [45]). Importantly, cervical contractions during the receptive phase (i.e. estrus) might be important for reproduction, helping to drive sperm into the uterus [36], [46].

Voltage-dependent K^+^ channels including Kv7 are known to play a key role in uterine pacemaking, whereas large conductance Ca^2+^ activated K^+^ channels (BK_Ca_) have little if any role [37], [38]. The fact that application of TEA, a blocker of voltage-dependent K^+^ channels, induced phasic contractions in previously quiescent mouse vaginal smooth muscle indicates that such channels also subserve a key role in vaginal pacemaking. Previous studies on rat [47] indicate that isolated resting vaginal smooth muscles are quiescent, though studies on isolated rabbit proximal vagina indicate sporadic spontaneous phasic contractions [48]. The fact that 1 of 22 mouse vaginal tissues was spontaneously active and that 10 mM TEA was able to induce phasic contractions in 19 of the other tissues suggests that there is underlying electrical pacemaker activity in these tissues under resting conditions (e.g. no neural input), but due to shunting of current by key K^+^ channels, pacemaker activity is generally unable to depolarize the smooth muscle to threshold until these channels are blocked.

Oxytocin (1 nM) induced an initial tonic contraction of vaginal smooth muscle, which reverted to phasic contractions during washout. Oxytocin is known to induce both tonic and phasic contractions in isolated uterine [49] and cervical [50], [51] smooth muscles. Agonists applied to middle and upper regions of the rabbit vagina can induce strong phasic contractions, which for the rabbit vagina occurred with exposure to norepinephrine and related agonists [48] or arginine vasopressin [52]. Human vaginal smooth muscle strips from women undergoing surgery to correct prolapse were quiescent and showed sustained contractions to carbachol and endothelin-1, agonists known to increase synthesis of inositol 1, 4, 5-trisphosphate (IP_3_) [47], [53]. In contrast, the human vagina has been reported to exhibit phasic contractions *in vivo* with response frequency and amplitude increased by balloon-induced distension [30]. In rodents vaginal contractions can be evoked as part of the urethrogenital reflex [1] indicating that vaginal phasic contractions *in vivo* are primarily under neural control, presumably via autonomic motor nerves entering the vagina via the pelvic nerves [35], [54]. Together, these findings suggest that during sexual activity, nerve release of agonists such as noradrenaline [48], associated distension [30] and released hormones such as oxytocin [46] will increase pacemaker effectiveness until threshold for opening voltage-activated L-type Ca^2+^ channels is reached and phasic vaginal contractions are induced.

### c-Kit or vimentin-immunoreactive ICs are unlikely to pace cervical and vaginal smooth muscles

It is well established that c-Kit and vimentin-immunoreactive ICCs drive spontaneous contractions in the gastrointestinal tract where there are extensive networks of ICCs associated with smooth muscle. C-Kit and vimentin-immunoreactive ICs are also present in uterine smooth muscle though at lesser density ([8]; see also Fig. 5B, F). However, the role of ICs in uterine pacemaking is unclear especially since electrophysiological recording from isolated uterine ICs did not exhibit inward ionic currents [8]. Inward currents are essential for pacemaking, as they are necessary to depolarize and trigger action potentials in the smooth muscle. Candidacy of c-Kit and Vimentin immunoreactive ICs as pacemakers in the cervix seems unlikely due to the their extremely sparse distribution and that they do not form networks. Thus even if these generated inward current, it is unlikely they would produce sufficient current to pace cervical smooth muscle. As we could find no evidence for such ICs associated with vaginal smooth muscle then these could not pace vaginal contractions.

Spontaneous activity in ICCs and resultant rhythmic electrical slow waves in the gastrointestinal tract are impaired by inhibitors of intracellular SERCA-dependent Ca^2+^ stores [21], [22], though the extent of such inhibition is dependent on the ICC subtype. For example, SERCA store inhibitors abolish pacemaker activity in gastric ICC-IM [20], [55], [56] but only partially in gastric ICC-MY with only the plateau component inhibited [57]. The reason for the latter finding is that ICC-MY have both an initial voltage-dependent component with T-type Ca^2+^ channel characteristics and a plateau IP_3_-store mediated pacemaker component [57]. By comparison, spontaneous cervical and TEA-induced vaginal contractions were not significantly altered during inhibition of the SERCA suggesting that pacemaking is solely mediated in these tissues by a voltage-dependent mechanism and but not one involving conventional SR/ER Ca^2+^ stores. The finding that contractions were inhibited by nifedipine parallels that for uterine smooth muscle where contractions arise through action potentials generated by L-type Ca^2+^ channels [58], [59], [60], though these are not pacemaker channels. However, while not tested, it remains possible that T-type Ca^2+^ channels may have a pacemaker role, as has been shown in pregnant rat uterine smooth muscle [59].

A surprising finding was that muscle tone did not increase upon application of CPA in both non-pregnant and pregnant mouse cervical and vaginal smooth muscle strips whereas CPA caused a large transient increase in tone in corresponding mouse uterine strips (Figure 6). An interpretation of these differences could be that there are minimal SR Ca^2+^ stores in cervical and vaginal muscle in comparison to the well-characterized stores in uterine muscle [13]. However, the finding that application of oxytocin, which is known to cause store Ca^2+^ release through activation of IP_3_ receptors [15], had a large effect on all three smooth muscles (Figure 4D–F) goes against this. This difference remains to be explored. Another observation made was that CPA caused the abolition of contractions in pregnant uterine smooth muscle. This could be due to large depolarization and inactivation of action potentials rather than a depletion of Ca^2+^ stores [16].

Despite a lack of evidence for c-Kit and vimentin immunoreactive ICs in driving phasic contractions in the FRT we do not rule out their involvement in modulating such activity in the cervix. Indeed in mutant mice showing reduced c-Kit labelling, uterine contractions are reduced in frequency but not abolished [12]. The behaviour of other reproductive tissues from these mutant mice has not been tested. The paucity of ICs in mouse vagina is in contrast with the findings for human vagina where ICs were present [61], [62] highlights the fact that further research on a range of mammals is necessary to understand the importance of these cells and this apparent species variation.

### Concluding remarks

Mouse cervical smooth muscles exhibit spontaneous contractions *in vitro* when taken from mice in late stage pregnancy and from non-pregnant mice during estrus and metestrus. Vaginal smooth muscle as studied *in vitro* was normally quiescent for all these conditions but exhibited phasic contractions when stimulated by the K^+^ channel inhibitor TEA or oxytocin. As for the uterus, SERCA-dependent Ca^2+^ stores do not appear to subserve a pacemaker role in cervical and vaginal smooth muscle. Vimentin and c-Kit-IR ICs, which are known to be present in the uterus, are minimally present in the cervix and were not found in the mouse vagina and hence are unlikely to have a primary role in pacemaking. Vaginal smooth muscle while quiescent at rest can readily be induced to exhibit phasic contractions by oxytocin, a hormone that is increased by sexual stimulation.
